# Overview of the Clinical and Molecular Features of Legionella Pneumophila: Focus on Novel Surveillance and Diagnostic Strategies

**DOI:** 10.3390/antibiotics11030370

**Published:** 2022-03-09

**Authors:** Giuseppe Gattuso, Roberta Rizzo, Alessandro Lavoro, Vincenzoleo Spoto, Giuseppe Porciello, Concetta Montagnese, Diana Cinà, Alessia Cosentino, Cinzia Lombardo, Maria Lina Mezzatesta, Mario Salmeri

**Affiliations:** 1 Department of Biomedical and Biotechnological Sciences, University of Catania, 95123 Catania, Italy; peppeg9305@gmail.com (G.G.); rroberta342@gmail.com (R.R.); alessandrolavoro@ymail.com (A.L.); leo_spoto@outlook.it (V.S.); alessiacosentino93@libero.it (A.C.); cinzialombardo@hotmail.com (C.L.); mezzate@unict.it (M.L.M.); 2 Epidemiology and Biostatistics Unit, National Cancer Institute IRCCS Fondazione G. Pascale, 80131 Naples, Italy; g.porciello@istitutotumori.na.it (G.P.); c.montagnese@istitutotumori.na.it (C.M.); 3 Health Management of the “Cannizzaro” Emergency Hospital of Catania, 95126 Catania, Italy; dianacinact@gmail.com; 4 Clinical Pathology and Clinical Molecular Biology Unit, “Garibaldi Centro” Hospital, ARNAS Garibaldi, 95123 Catania, Italy

**Keywords:** *Legionella pneumophila*, surveillance strategies, diagnostic strategies, antibiotic resistance, virulence factors

## Abstract

*Legionella pneumophila* (*L. pneumophila*) is one of the most threatening nosocomial pathogens. The implementation of novel and more effective surveillance and diagnostic strategies is mandatory to prevent the occurrence of legionellosis outbreaks in hospital environments. On these bases, the present review is aimed to describe the main clinical and molecular features of *L. pneumophila* focusing attention on the latest findings on drug resistance mechanisms. In addition, a detailed description of the current guidelines for the disinfection and surveillance of the water systems is also provided. Finally, the diagnostic strategies available for the detection of *Legionella* spp. were critically reviewed, paying the attention to the description of the culture, serological and molecular methods as well as on the novel high-sensitive nucleic acid amplification systems, such as droplet digital PCR.

## 1. Introduction

*Legionella* species (spp.) are Gram-negative, aerobic, rod-shaped, non-spore-forming bacteria which are ubiquitous in water environments, such as lakes, rivers, hot springs, and ponds, as well as in composted materials and moist soil. The genus *Legionella* currently includes 66 species and more than 70 serotypes, half of them recognized as opportunistic pathogens for humans [1].

*Legionella* takes its name from an outbreak of pneumonia occurring in Philadelphia during a conference of U.S. military veterans in 1997. During this conference, 221 veterans of the American Legion contracted *Legionella* infection via water transmission. Of these, 34 veterans died highlighting for the first time the health problem represented by *Legionella* outbreaks [2]. A year later, *Legionella pneumophilia* (*LP*) was recognized as the etiologic agent of Legionnaires’ disease (LD) [3]. Currently, 15 different serogroups of LP were recognized of which serogroup 1 is the most clinically relevant accounting for 80–90% of Legionnaires’ disease cases, while other serogroups (2–15) are less common and occasionally cause legionellosis.

Other etiological agents of legionellosis are *Legionella longbeachae*, *Legionella micdadei*, *Legionella bozemanii* and *Legionella dumoffii* [4,5]. In particular, *L. longbeachae* is the causative agent of approximately 30% of community-acquired LD in New Zeeland and Australia [6], while *L. micdadei* represents the second most common cause of LD in the United States and Europe [7].

As widely known, *Legionella* spp. colonizes freshwater and moist soil environments as intracellular bacteria, infecting and replicating inside eukaryotic cells, such as free-living amoebae, monocytes and alveolar macrophages. In this way, *Legionella* is protected from different chemical and physical agents, thus developing several resistance mechanisms [8]. Frequent hosts of *Legionella* spp. are different amoebae including *Acanthamoeba*, *Naegleria*, *Balmuthia*, *Dictyostelium* and ciliate cells, such as *Tetrahymena* [9]. It has also been demonstrated that *Legionella* spp. Are naturally part of microbial ecosystems associated with complex biofilm communities, where they can survive in water systems [10].

Although *Legionella* spp. Are in low concentrations in natural environments, they can increase significantly in artificial water systems, where conditions are favorable for their proliferation due to the presence of biofilms and temperatures ranging between 25 °C and 45 °C, with a gold-standard temperature of 37 °C [10,11].

In the last few years, the incidence of *Legionella* infection has increased in both the United States and Europe. According to the reports from the *European Centre for Disease Prevention and Control*, in 2019, the overall notification rate in the EU/EEA was 2.2 cases per 100,000 inhabitants. Specifically, the annual rate from 2015 to 2019 has increased from 1.4 to 2.2 cases per 100,000 population, with the highest rate reported in Slovenia (9.4 cases per 100,000 population) [12].

Noteworthy, the incidence of legionellosis in Italy has increased over the years, reaching 52.9 cases per million inhabitants in 2019, which shows a slight increase compared to the previous year (48.9/1,000,000) [13].

During the last two years, some studies reported an opposite trend in the notification rate of *Legionella* infections compared with the above-mentioned data. Specifically, Fischer and colleagues evaluated the trends of legionellosis from 2000 to 2020 in Switzerland and other European countries. The highest notification rate was recorded in 2018 with 6.7/100,000 cases, while the trend after COVID-19 restriction measures showed a temporary decrease of 35%. A recent report by the Federal Office of Public Health (FOPH) showed also a reduction of LD cases of 32% compared with the expected case numbers observed from 2015 to 2019 [14]. As already mentioned, 52.9/100,000 cases of legionellosis were reported in Italy in 2019, while preliminary data for 2020 show a 35% decrease in the number of reported cases [15]. Such a decrement in the notification rate of legionellosis was surprising as the water tanks and pipes were not properly monitored due to the lock-down restrictions, therefore, an increment of *Legionella* exposure and infection was expected [16]. Recently, De Giglio and colleagues revealed that the water systems of some hospital wards showed a higher *L. pneumophila* contamination due to the 3-month closure during the COVID-19 emergency. In addition, Liang and colleagues and Chao and Lai described the possible increased risk of *Legionella* exposure after the end of lock-down [17,18]. Another possible explanation for the low notification rate observed during the COVID-19 pandemic could be related to similar symptoms existing between SARS-CoV-2 and LP pneumonia; therefore, an underestimation of LD pneumonia could have occurred during the pandemic [14,15].

The epidemiological data mentioned above suggest that the incidence of legionellosis and its associated health risks are expected to keep increasing due to global challenges, such as urbanization, climatic changes and new economic approaches. Therefore, the improvement of techniques and strategies for the environmental surveillance and management of *Legionella* in critical environments, such as health care structures and long care facilities is one of the most important challenges for the prevention of legionellosis.

On these bases, the present manuscript aims to provide a broad and updated description of past and current strategies implemented for the management of LP in healthcare environments. To the best of our knowledge, this review represents an advancement of current knowledge on LP diagnosis and treatment providing an updated description of novel treatments, water disinfection methods and molecular diagnostic techniques as widely discussed in the following chapters.

## 2. *Legionella* Clinical and Molecular Features

Among *Legionella* spp., *Legionella pneumophila* is the most implicated in human infections and responsible for different clinical manifestations: Legionnaires’ disease, severe pneumonia that can lead to permanent lung damage or death, Pontiac fever, a milder influenza-like disease, and rarer forms of extrapulmonary infection [19,20].

Patients’ risk factors associated with LD include older age, male sex, smoking, chronic lung diseases, cardiovascular diseases, or renal disorders [21].

The most common clinical symptoms of both LD and Pontiac fever are cough, muscle ache, headache, and shortness of breath. Fever and fatigue may precede the onset of cough. In LD, the symptoms usually appear after 2–14 days of incubation, while symptoms of Pontiac fever occur a few hours to three days after being exposed to the bacteria and typically last less than a week [22].

The main clinical feature of LD is pneumonia, which is clinically and radiographically characterized by irregular, unilobular infiltrates, which may progress to permanent lesions [23]. Signs and symptoms of lower respiratory tract infections are absent in Pontiac fever. Moreover, LD can also be associated with gastrointestinal symptoms, such as diarrhea, nausea, and vomiting [22].

Regarding LD, it was widely demonstrated that serious complications, including lung failure or death, may occur. In some cases, the presence of rare extrapulmonary complications, including cellulitis, skin abscesses, septic arthritis, endocarditis, meningitis, and peritonitis, was observed in immunocompromised patients [24,25,26].

It has been also described that LD mortality rate ranges between 1 to 10%, while Pontiac fever have usually a benign course without requiring any specific treatments [27,28].

From a clinical point of view, *Legionella* infections are divided into community-acquired and nosocomial infections. The second one can be acquired in health care settings during hospitalization. The nosocomial infections arise at least 48 h after admission, during the hospital stay, or after discharge, while community-acquired infections are already present at the time of admission [29].

After infection, *LP* grows in alveolar macrophages of human lungs using an escape mechanism resulting from the evolution of an opportunistic infection mechanism which *LP* uses in parasitized protozoa [30]. This mechanism consists of bypassing the canonical bactericidal endocytic pathway and forming an ER-associated replication-permissive compartment, called *Legionella*-containing vacuoles (LCV) [31,32].

Remarkably, many studies have shown that the *LP* genome contains several virulence genes involved in the entire infection cycle, which have been described as the most important factors affecting the ability of *Legionella* to grow and survive in alveolar macrophages and in free-living amoebae [33]. These virulence factors, termed pathogenicity island locus (PAIs) and encoded by specific DNA regions in the genome of pathogenic bacteria, are associated with *Legionella* pathogenicity while the same genes are not present in non-pathogenic strains [33,34]. Accordingly, an important role in the pathogenicity of *Legionella* is played by structures of the cell surface [35]. Specifically, adherence and subsequent invasion of the bacterium into the alveolar macrophages and protozoa are favored by the expression of some surface proteins.

The most abundant surface protein synthesized by *LP* is a 60-kDa heat shock protein (Hsp60) encoded by the high temperature protein B (*htpB*) gene. This protein modulates macrophage function via a mechanism that involves surface interactions, enhancing invasion and cytokine expression in macrophages in the pathogenesis of LD [36].

Of note, recombinant major outer membrane protein (MOMP), a protein encoded by the *mompS* gene, plays an important role during the attachment of *Legionella* to host cells. Specifically, this protein mediates the activation of alternative pathways of Complement Receptor 1 (CR1) and Complement Receptor 3 (CR3), leading to phagocytosis of *LP* by human monocytes [37].

Another protein associated with the virulence of *LP* is a 24 kDa protein encoded by the macrophage infectivity potentiator (*Mip*) gene which increases *LP* entrance in macrophages. Mip is exposed on *Legionella’s* surface and is involved in cell penetration. Interestingly, Mip is known to be one of the first genes associated with the ability of *LP* to replicate in eukaryotic cells [38].

Other important structures that play a crucial role in the infection process of *Legionella* are the secretion systems. It is widely known that many pathogenic bacteria use specialized protein secretion systems to introduce virulent effector proteins or other factors into host cells.

Specifically, *Legionella* is able to control the formation of LCVs and other pathogen-host interaction structures through the secretion of several proteins. Among the secretion systems, the putative type I Lss secretion machinery, type II PilD-dependent Lsp, type IVA lvh, and type IVB Icm/Dot secretion pathways are those involved in *Legionella* infectivity [39].

The *LP* type I Lss secretion system (T1SS) is responsible for the secretion of the repeats-in-toxin protein rtxA, which contributes to cellular entry and subsequent attachment to host cells (D’Auria G et al., 2008). rtxA is also involved in the intracellular survival and trafficking of the bacteria [34]. Particularly, the locus encoding for T1SS, named lssXYZABD, includes the typical components of a type I secretion system, including an ATP-binding cassette transporter (LssB) and a membrane fusion protein (LssD) [40]. Interestingly, all *LP* strains described in the study of Qin and colleagues contained the lssXYZABD locus. In contrast, the lssXYZABD locus was not found in non-*L. pneumophila* species, suggesting that the lssXYZABD secretion system plays an important role in *LP* biology [41].

*LP* also has a PilD-dependent type II secretion system (T2SS), termed Lsp, which is involved in the *Legionella* secretion pathway. This secretion system is required for *Legionella* virulence and environmental persistence. It has also been demonstrated that *LP* T2SS Lsp promotes intracellular infection of lung epithelial cells, attenuates cytokine secretion from infected macrophages and epithelia, and limits the number of cytokine transcripts in infected macrophages [42].

Type IV secretion systems (T4SSs) have been divided into two subclasses: type IVA (T4ASS), which resembled the Vir system of *Agrobacterium tumefaciens*, and type IVB (T4BSS), which is comparable to the Tra/Trb bacterial conjugation systems [39]. Specifically, the secretion systems type IVA lvh and type IVB Icm/Dot are present in *LP*.

In particular, the *Legionella* vir homolog (*lvh*) locus forms the protein for a second type IV secretion system that contributes to conjugation, virulence, and survival of the bacteria in the environment [43]. Previous studies have reported that the *lvh* and *rtxA* regions are more abundant in strains associated with human disease [44,45,46].

The gene cluster encoding T4ASS, referred to as *lvh*, is located in a region with high GC content, which allows the mobility of *lvh* region in the genus *Legionella*, contributing to the interspecies exchange of genetic information [47].

Once again, in the study of Qin and colleagues, the *lvh* genes were found in seven non-*L. pneumophila* and 40 *LP* strains, showing that the sequence of T4ASS *lvh* genes is highly conserved in these strains [41].

The type IVB secretion system (T4BSS) is termed Icm/Dot (Intracellular multiplication/defective organelle trafficking) and is a conjugation system used for the transport and injection of DNA or toxins into target cells [48,49,50].

In *LP*, the T4BSS is encoded by two separate pathogenicity regions on the chromosome. The first region contains 17 genes (*icmTSRQ-PONMLKEGCDJBF*), while the second region contains *icmXWV* and *dotABCD* [51].

Alveolar macrophages appear to be the primary cell type that is targeted by T4SS effectors and support intracellular bacterial replication [52]. Moreover, T4SS enables *LP* to manipulate a variety of cellular processes, including membrane trafficking, protein synthesis, ubiquitylation and autophagy [53,54,55,56].

Two-component systems (TCS), also known as two-component response regulators, are widespread signal transduction devices in bacteria that allow them to respond to environmental stimuli mainly via changes in gene expression. These systems are used by many pathogenic bacteria, including *Legionella*, to control the expression of their virulence genes [57]. In particular, the CpxRA TCS consists of the sensor histidine kinase (CpxA) and the cytoplasmic response regulator (CpxR). It recognizes various envelope stressors and promotes the transcription of genes that helps bacteria to overcome these stressors [57].

Of note, CpxRA is involved in the regulation of Dot/Icm system components and effectors. CpxR has been shown to directly activate the expression of the four Dot/Icm system components *icmR*, *icmV*, *icmW* and *lvgA* and to activate and repress 11 translocated effector proteins [58].

From these perspectives, the identification of particular virulence factors or drug resistance genes could be useful to clinicians to administer the most effective treatment and predict the prognosis of patients.

The most important virulence factors and their corresponding encoding genes are summarized in Table 1.

## 3. Pharmacological Treatment of Legionellosis and Management of *LP* Resistant Clones

The clinical manifestations of *Legionella* infections range from benign, mild disease to a more severe form with increased morbidity and mortality, especially in untreated patients. As the clinical and radiological symptoms of *Legionella* infection are nonspecific, empiric antibiotic treatment, effective towards a broad spectrum of common pneumonia pathogens, is recommended in the case of a suspected *LP* infection in order to reduce the morbidity and mortality associated with this disease [61,62].

Current American and European guidelines recommend macrolides and fluoroquinolones (azithromycin and levofloxacin, respectively), as first-line treatments for severe and moderate LD [63]. In detail, according to the British Thoracic Society (BTS) recommendations, the administration of 500 mg azithromycin or levofloxacin (500 mg IV/d) every 24 h is recommended in the case of mild LD pneumonia. In the presence of patients with severe LD pneumonia or in case of drug resistance, the second-line treatment is based on the combination of levofloxacin (500 mg IV/d) or another fluoroquinolone plus azithromycin (500 mg IV every 24 h) [64].

In a recent study by Miyashita and colleagues, it was observed that fluoroquinolones and macrolides have potent antimicrobial activity against both extracellular and intracellular *Legionella* spp., suggesting the good efficacy of these drugs in the treatment of *Legionella* infections [65].

Specifically, macrolides are bacteriostatic agents that bind reversibly to the 50S ribosomal subunit and inhibit protein synthesis [66]. They are effective against a wide range of bacteria, including intracellular pathogens. Macrolides, especially azithromycin, reach their peak concentration within 2–3 h and are rapidly absorbed and distributed throughout body tissues and cells [66].

In addition to macrolides, fluoroquinolones (levofloxacin, moxifloxacin, and ciprofloxacin) have increasingly become the standard treatment for *Legionella* infection, because they have a broad spectrum of activity against Gram-positive and Gram-negative organisms [67].

In particular, fluoroquinolones inhibit DNA gyrase subunit A blocking the transcription of bacterial DNA, resulting in bacterial cell death. Additionally, some studies recently reported that fluoroquinolones could be superior to macrolides due to their broad spectrum of activity and fewer adverse effects [68,69,70].

Despite the efficacy of macrolides and fluoroquinolones, treatment failures have been recently reported in the literature, indicating the possibility of developing resistance to traditional therapies [71]. In this context, Bakheit and colleagues have described some general mechanisms implicated in multiple bacterial resistance to macrolides. One mechanism is an active efflux pump, which ejects the drug from the bacteria cell. At least 16 different genes have been identified in connection with this mechanism. Another resistance mechanism involves some modifications in the ribosomal subunits, which reduce the binding of the antimicrobials to the ribosomal target site [71].

Regarding *Legionella*, mutations in genes encoding 23S rRNA or ribosomal proteins are known to be responsible for macrolide resistance in *LP* strains [71]. In particular, Descours and colleagues focused on mutations affecting key determinants of reduced susceptibility to macrolides, such as genes encoding 23S rRNA (*rrl*), L4 (*rplD*), and L22 (*rplV*) ribosomal proteins. The results highlighted that an initial mutation on ribosomal L4/L22 proteins causes moderately reduced sensitivity to macrolides and additional mutations in genes encoding 23S rRNA are responsible for an increased resistance [71].

In addition, some studies have demonstrated that the presence of the *lpeAB* genes and the efflux pump component *lpeAB* are associated with reduced susceptibility to azithromycin [72,73,74,75]. In particular, Massip and colleagues showed that *lpeAB* genes encode components of a trimeric efflux pump responsible for resistance to azithromycin among other macrolides while Vandewalle-Capo and colleagues demonstrated that the reduced sensitivity to azithromycin in ST1 strains was linked to the presence of *lpeAB* genes. More recently, Jia X and colleagues have tested 25 strains of *Legionella*, showing how the expression of *lpeAB* was responsible for reduced azithromycin susceptibility in all these strains. Similarly, Cocuzza and colleagues demonstrated the role of *lpeAB* efflux pump system in *LP* isolates which reduces susceptibility to azithromycin [73].

Additionally, fluoroquinolone-resistant *Legionella* strains have recently been identified in patients treated with these agents [76]. Notably, it has been described that bacterial resistance to quinolones is the result of chromosomal mutations of the DNA gyrase gene, leading to a decreased affinity of the drug for the enzyme. Similar to macrolides, alterations in drug efflux may also occur, resulting in a lower intracellular concentration of the drug [77].

Some researchers have also demonstrated that *LP* fluoroquinolone resistance in tested strains is associated with mutations affecting the type II topoisomerase-encoding genes (i.e., DNA gyrase (*gyrA* and *gyrB*) and topoisomerase IV (*parC* and *parE*)). Among these, *gyrA* is the primary target of fluoroquinolone in *LP*. When mutations affect *gyrA*, the susceptibility of DNA gyrase towards fluoroquinolones is reduced. Another target of fluoroquinolones is *parC* gene involved in the formation of topoisomerase IV. Consequently, the presence of additional mutations on *parC* is responsible for a stronger reduction of *LP* susceptibility to fluoroquinolones [77].

Recently, Hennbique and colleagues detected mutant *gyrA* sequences in mixtures of fluoroquinolone-resistant and susceptible *LP* strains by using digital PCR (dPCR) systems. These data suggest that dPCR allows rapid and accurate detection and quantification of these resistant mutants in respiratory samples [78].

To overcome these drug resistance mechanisms, recent studies focused their attention on the development of new therapies for the treatment of resistant *LP*. Among the most promising therapies, antisense therapy can eliminate or re-sensitize pathogens by targeting the *LP* vesicle trafficking pathway. Specifically, antisense therapies mediate the intracellular trafficking pathway to prevent the fusion of phagosomes and lysosomes in macrophages. In this way, bacteria in lysosomes are effectively killed by lysosomal enzymes [79]. Other current studies are evaluating the therapeutic potential of recombinant DNA vaccines against some virulence factors, such as peptidoglycan-associated lipoprotein (PAL) and PilE. In particular, an increased and stronger cellular and humoral immune response has been observed in mice after vaccine administration which resulted in a faster remission of the infection [80,81].

All these data suggest that it is important to analyze *Legionella* isolated, especially in hospital water systems, in order to early detect changes in antibiotic resistance patterns thus preventing potential outbreaks caused by antibiotic-resistant bacteria.

## 4. Monitoring of *Legionella* spp. in Hospital Environments and Water Disinfection Strategies

As stated by some recent reports, legionellosis represents a public health problem, which is escalating rapidly worldwide [82,83,84].

Of note, the main reservoirs for *Legionella* spp. are represented by water distribution systems, especially in large public buildings, households and industrial facilities [85]. However, the most dangerous type of colonization occurs in water distribution systems, cooling towers and hydric pipeline of hospitals, because in these environments *LP* can multiply causing severe infection in immunosuppressed hospitalized patients. Indeed, the most common route of exposure is represented by the inhalation of aerosol droplets containing *Legionella*, and the risk of transmission increases when the complexity of hospital water systems and patients’ susceptibility are considered [86]. Despite human-to-human transmission of legionellosis being very unlikely, a case was recently documented [87].

According to the latest available data from the European Centre for Disease Prevention and Control (ECDC), 10,672 confirmed cases of Legionellosis were reported in 2018, of which 6% were hospital-acquired. Specifically, in Italy, there were 3199 cases of legionellosis in 2019, of which 3.8% were hospital-acquired cases [88].

On these bases, environmental monitoring of *LP* in hospitals is a useful strategy to prevent nosocomial LD [89]. In order to prevent and reduce *Legionella* colonization and nosocomial cases of legionellosis, national and international guidelines started recommending environmental monitoring and remedial measures to control *Legionella* spp. contamination in water. In line with the World Health Organization (WHO), one of the best approaches to assess the health risks associated with *Legionella* colonization is the implementation of a water safety plan (WSP) [90].

According to the WHO recommendations, WSPs are based on the risk analysis methodology, a process for identifying hazardous events and determining the risks associated with the occurrence of *Legionella* spp. contamination. Specifically, WHO recommendations promote the assessment of risks and the development of measures for their control. Briefly, an effective water safety plan describes and monitors water systems, identifies potential hazards, assesses and prioritizes potential risks, identifies and implements control measures to mitigate the risk, defines corrective actions and verifies water system managing programs [91].

**Figure 1:**
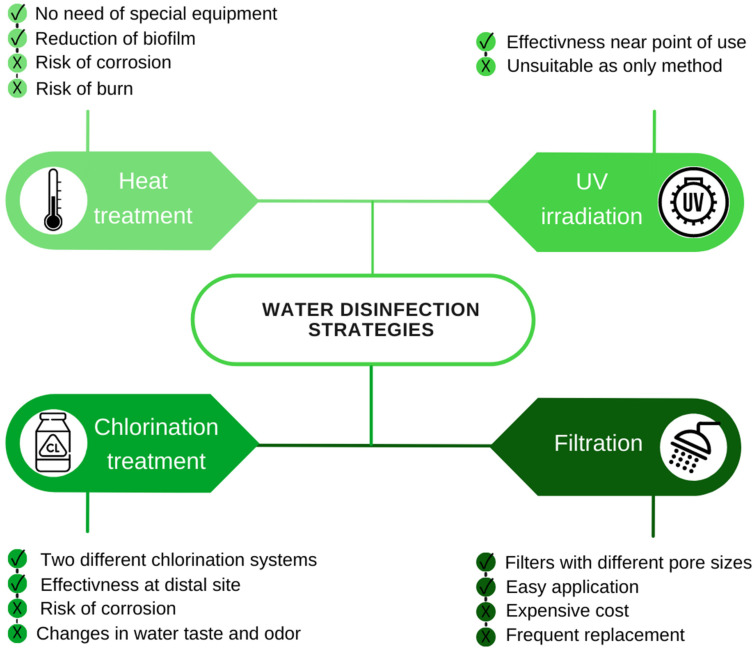
Pros (√) and cons (×) of the mainly adopted water disinfection strategies.

Different treatments are currently available for the disinfection of water. These treatments can be physical (heat treatment and ultraviolet irradiation) or chemical (use of metal ions and oxidizing agents) [12] (Figure 1).

Heat treatment consists in raising the water temperature to at least 60 °C in order to inactivate *Legionella*. Currently, two main heat treatments are used. In the first method, the thermal shock is performed by maintaining the temperature of the water between 60 °C and 80 °C for three days. For effective disinfection, hot water at 60 °C must flow in all pipes for at least 30 min during the three-day treatment. In the second method, after a first heat-shock treatment at 70 °C, it is possible to keep the temperature at 55–60 °C at the distal points. Numerous public structures have adopted this technique because it does not require special equipment and it is also capable of reducing biofilms and eliminating *Legionella* colonies. However, heat treatments are not always applicable due to the high temperatures which lead to the corrosion of pipes, significant energy consumption and risk of burns [92].

Ultraviolet (UV) light irradiation has shown great efficacy in eradicating *Legionella* [93]. In particular, UV acts on bacterial DNA producing thymine dimers that inhibit DNA replication. Due to the lack of residual power, UV irradiation alone is not sufficient to control the presence of *Legionella* in the system. Other limits related to the use of UV are the presence of biofilms, the turbidity of the water and sediments which could act as a shield to the radiation. Therefore, this technique is not suitable as the only method for a whole building, because it does not have a residual effect, but is more effective near the point of consumption [93].

Filtration is a physical method based on the use of filters with pores of 0.22 µm placed at the points of use (POU), such as taps and showers. Filters are usually in hospitals to protect patients and medical staff in vulnerable departments. There are several different filtration systems that can remove contaminants: ultrafiltration, microfiltration, nanofiltration, and reverse osmosis processes. The disadvantage lies in the cost and frequent replacement, especially for hard water. Additionally, microbial filtration systems applied to POU require high water pressure to achieve the desired flow rate. Filters with small pore sizes need higher applied pressure than filters with large pore sizes [94].

Another physical method involves the use of ionization chambers with copper-silver electrodes installed on water pipes. Copper and silver have a bactericidal effect through the ionization of the cell wall of the microorganism which causes an alteration of cell permeability. In addition, both ions interfere with cellular respiration leading to cell lysis and death [95].

Interestingly, the work of Cloutman-Green and colleagues shows that it is possible to control *LP* incidence in a new hot water network at low temperatures (room temperature) using copper-silver ionization [96]. Therefore, ionization represents an easy-to-use method that is not affected by water temperature and its bactericidal effect can persist for several weeks thanks to the accumulation of copper in the biofilm. However, it has the disadvantage of being unsuitable for zinc pipes, as this metal causes the inactivation of silver ions. The use of ions requires constant maintenance of the electrodes and a careful evaluation of the doses depending on the characteristics of the system.

A similar method is based on the use of hydrogen peroxide and silver salts, which act synergistically with a complementary mechanism capable of radically destroying the protein material of the biofilm, penetrating deeply and inactivating microorganisms, including *Legionella*. It is a preventive measure that can be used as an alternative to heat or chlorine treatment [97].

Among the chemical methods, chlorination, chloramination and chlorine dioxide (CLO2) purifications are chemical processes that increase residual chlorine in water, resulting in the formation of toxic byproducts (chloroform, trihalomethanes bromoform, dibromochloromethane and bromodichloromethane) [94,98].

Chlorine is available in gaseous form and in the form of sodium or calcium hypochlorite. Although chlorination is the most used chemical method, it must be applied continuously in water tanks. In particular, there are two different methods of chlorination: intermittent and continuous.

The first method, also known as shock hyperchlorination, involves a single injection of chlorine into the water for 1–2 h until a high concentration (20–50 mg/L) of free residual chlorine is reached throughout the system, including distal sites. The other method involves a continuous injection of chlorine (0.5–1.0 mg/L) in the form of calcium or sodium hypochlorite. This method ensures a residual chlorine concentration throughout the water system, minimizing *Legionella* colonization at distal sites [98].

In this context, Paranjape and colleagues and Mouchtouri and colleagues showed that the effect of chlorination in cooling towers in Canada and Greece was crucial to minimizing the colonization and recolonization of *Legionella* spp. [99,100].

However, the significant disadvantage of hyperchlorination is the relative inability to eradicate the organism from the water distribution system, facilitating potential recontamination. In addition, strict and continuous monitoring of chlorine levels is required for the effectiveness of this method. Furthermore, chlorine has a significant corrosive effect on the water distribution pipes and can cause a change in the taste and odor of the water [101].

Similar to chlorination, chloramination is characterized by the formation of monochloramine, a weaker oxidant than chlorine but more effective and stable than chlorine dioxide [102]. Monochloramine has also a significant anti-*Legionella* activity and long-term efficacy, especially in complicated pipe networks [98]. The use of this chemical produces an excess of ammonia and a minor chloramine residue, due to the potential of monochloramine to react with organic substances in water creating byproducts. Additionally, rubber and plastic parts used in water systems have also been observed to be affected by chloramine [102].

Another chemical disinfection method is based on the use of chlorine dioxide (ClO_2_), an antioxidant that kills waterborne pathogens and associated biofilms. In addition, ClO_2_ is excellent in controlling the taste and color of drinking water. However, one disadvantage of using ClO_2_ in *Legionella* control is the potential corrosion of iron pipes [103].

Finally, ozone injected into water is another chemical method that has been used to control *Legionella* in building water systems [104]. It acts quickly by damaging bacterial DNA, and it is more effective than chlorine. On the other hand, it has no residual power and has limited effectiveness because it does not penetrate biofilms. In concentrated form, it can damage pipes. Its high cost and the need for specialized maintenance personnel make it an effective but not a commonly used method [98].

More recently, novel methods for the inactivation of *LP* in drinking water have been developed using LED emitting UV-C rays. In particular, LED UV-C irradiation of drinking water at 255 nm and 0.5 mJ/cm^2^ of fluence effectively reduced the log of all the tested *LP* serogroups [105]. These further data encourage the adoption of LED at POU due to its low cost of production and durability.

## 5. Detection of *Legionella pneumophila* and Diagnosis of Legionellosis

Specific detection methods have been developed for the diagnosis of *LP* infection using specimens obtained from the respiratory tract (e.g., sputum, bronchoalveolar lavage (BAL)) or liquid biopsy samples like serum or urine samples. The main diagnostic approaches include bacterial culture, serological and antibody-based assays, nucleic acid detection systems (e.g., polymerase chain reaction) and urine antigen tests [20].

Isolation by culture methods is considered the gold standard for the diagnosis of *Legionella* infections. The most used medium for the growth of *Legionella* spp. is BCYE (Buffered Charcoal Yeast Extract), which consists of CYE agar base supplemented with cysteine, iron salts and α-ketoglutarate in ACES (n-(2-acetamido)-2-aminoethanesulfonic acid) buffer, which ensures an optimal pH (pH 6.9) for the growth of *Legionella*. In this medium, the yeast extract serves as a source of nutrients, while activated carbon is used to eliminate various toxic compounds produced in the soil, especially after exposure to light, such as reactive oxygen species [106].

In the presence of contaminating microorganisms, heat and/or acid pre-treatments of waters samples should be performed. Particularly, the water samples can be treated with HCl pH 2.2 for 5–20 min or heated up to 50 °C for 30 min or 60 °C for 1–3 min depending on the levels of contamination [107].

Additionally, other selective media are used to isolate *Legionella* spp. from potentially contaminated clinical samples. The most commonly used selective media is MWY (Wadowsky-Yee Medium) and Glycine Vancomycin Polymyxin Cycloheximide (GVPC) medium. Both media contain polymyxin B, which inhibits the growth of Gram-negative bacteria, vancomycin which targets Gram-positive bacteria while glycine impairs the bacterial wall facilitating the action of antibiotics [1].

The MWY medium also contains azinomycin which acts against yeasts, while dyes give a characteristic color to certain *Legionella* species. The GVPC medium, instead, is enriched with cycloheximide which suppresses the growth of fungi but is highly toxic by contact and inhalation. In this regard, there is also the possibility to use a different formulation, called Glycine Vancomycin Polymyxin Natamycin (GVPN), which contains natamycin, a non-toxic antifungal as effective as cycloheximide [1].

Interestingly, Di Tommaso and colleagues evaluated the diagnostic performance of BCYE and MWY in 951 *Legionella*-positive water samples from hospital environments. It was found that MWY allowed the detection of *Legionella* in 89.2% of the samples. In particular, MWY is essential to detect Legionella in samples contaminated by multiple organisms (52.6%, 349/663).

In samples contaminated by *Legionella* only, a higher frequency of positive samples was recorded using BCYE (94.8%, 273/288) compared to MWY (85.1%, 245/288). These findings confirm the appropriateness of the ISO 11,731:2017 update encouraging the use of selective media for correct detection of *LP* [108].

Regarding the growth of *Legionella* on culture media, it forms colonies under microaerophilic conditions (2.5% CO_2_) showing considerable pleomorphism. Initially, the colonies are small and punctiform, however, after several days of incubation they increase in diameter and reach up to 3–4 mm. They appear circular shape in shape, and it is possible to distinguish a white central part and shiny grey-white clear margins with a mucous consistency. It is also known that *LP* colonies emit yellow-green fluorescence when observed under UV lamps [109].

All *Legionella* spp. can be detected with culture methods; thus, these represent the gold standard for the diagnosis of legionellosis. Unfortunately, culture methods have some critical issues, mostly represented by the low growth rate of *LP*, which requires long waiting times (often taking 5 days or more to grow). Moreover, only half of patients with LD produce sputum. Furthermore, the culture method is impractical in some cases because the growth of *Legionella* can be inhibited by the presence of other bacteria [110].

Besides culture methods, serological tests can be used to assess *Legionella* infection. Among these methods, indirect immunofluorescent assays (IFA) and enzyme-linked immunosorbent assays (ELISA or EIA) are the most frequently performed tests [111]. However, the use of these techniques has declined significantly, due to the development of faster and user-friendly methods, such as the urinary antigen test. Indeed, both IFA and ELISA required the collection of two serum samples at different time points lengthening the time of diagnosis. Despite this limitation, serological tests remain relevant for retrospective epidemiological investigations and when the infectious agent cannot be isolated despite clear evidence of LD [112].

Another detection method is direct fluorescent-antibody (DFA) staining, a rapid method for the direct detection of *Legionella* spp. in respiratory secretions and tissue samples. The limitation of this method is his poor sensitivity due to cross-reactions with other respiratory pathogens [12].

More recently, nucleic acid amplification systems have gained the attention of researchers and clinicians for their high-sensitive diagnostic value for different diseases. The current COVID-19 pandemic has demonstrated the high diagnostic value of different molecular methods, including quantitative reverse transcription PCR (RT-qPCR), droplet digital PCR (ddPCR), biosensors and other point-of-care systems, able to detect the pathogen in different types of samples [113,114,115]. In line with these recent findings in COVID-19, the polymerase chain reaction (PCR) has been used for the detection of *Legionella* in respiratory secretions (e.g., sputum or BAL) as well as urine and serum samples [116,117,118]. PCR has numerous advantages compared to other diagnostic methods. Some studies have reported that PCR is more sensitive than culture methods, suggesting that it is a useful tool for identifying sources of infections [119,120,121]. One of the most important advantages of this method is the rapidity of the results as PCR results can be available approximately 4 h after sample collection and are easier to interpret than *LP* culture, which requires a longer time (approximately 5–8 days) [121].

Remarkably, some studies have described that multiplex PCR is capable of simultaneously detecting and discriminating different *Legionella* species or serogroups. This method is preferably used in outbreaks or for surveillance strategies. The amplification of nucleic acids has many advantages. Firstly, PCR does not require previous culture isolations. Notably, it does not need the presence of living organisms, which is important during the detection of *Legionella*, especially in patients under pharmacological treatments with antibiotics [122,123,124]. However, these methods also detect nucleic acids from dead or dying bacteria, organisms associated with amoeba and viable but nonculturable (VBNC) microorganisms overestimating the real bacterial load.

In order to overcome these critical points, some researchers have proposed the use of a droplet digital PCR (ddPCR) system as a novel and time-saving method for the rapid detection of *Legionella* spp. [125,126,127].

Falzone and colleagues demonstrated the high sensitivity and accuracy of ddPCR compared to RT-qPCR in detecting *LP* in water samples with a low bacterial load. Specifically, RT-qPCR detected a low concentration of *L. pneumophila* at a very late Ct value, while ddPCR precisely quantified the concentrations of *L. pneumophila*. Of note, it was described that ddPCR is not affected by the presence of fragmented DNA, suggesting that this method can be used to detect the pathogen in patients with suspected legionellosis under antibiotic treatments.

Similarly, Logan-Jackson and colleagues used ddPCR to characterize the abundance of *Legionella* spp. and five specific *Legionella* species from samples (groundwater) obtained from exposed sites. Using ddPCR, the authors were able to quantify low DNA concentration in water samples as well as the abundance of DNA in a host-pathogen interaction context identifying the co-occurrence of pathogenic *Legionella* spp. (*L. pneumophila, L. anisa, L. longbeachae, L. bozemanii, and L. micdadei*) and amoebae species.

Although these studies suggested that ddPCR should be used for the monitoring of water samples, *Legionella* molecular tests need improvements before commercialization [128,129,130].

Finally, when PCR is not available or when sputum cannot be obtained, urine antigen testing represents a good diagnostic alternative. The sensitivity of urine antigen tests ranges from 70% to 80% with a 100% specificity [130]. *Legionella* antigens can be detected in urine the day after the onset of symptoms and persist for a couple of weeks. Even if the urine antigen test is negative but *Legionella* infection is suspected, it is reasonable to perform PCR or culture on a lower respiratory tract specimen [131].

**Figure 2:**
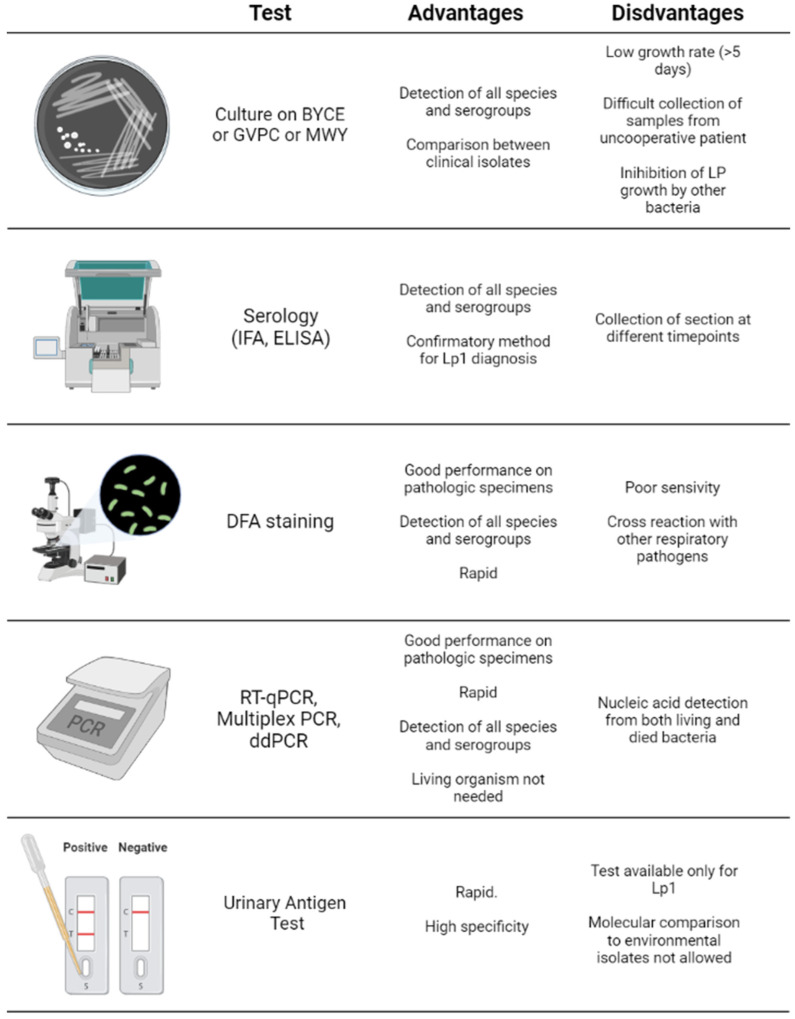
Advantages and pitfalls of the culture, serological and molecular methods for the diagnosis of legionellosis.

The main advantages of the urine antigen test are its rapid turnaround time and high specificity; however, this test can be used only for the detection of *LP* serotype 1 [132]. While *LP* serotype 1 causes over 80% of reported cases of LD in most regions of the world, *L. longbeachae* is widely distributed in some regions, such as Australia and New Zealand, limiting the utility of the urine antigen test [6]. However, antigen tests for the detection of *L. longbeachae* are under development [133] (Figure 2).

## 6. Conclusions

Despite the development of more accurate diagnostic methods and increasingly accurate water monitoring and sanitation strategies, *Legionella* spp. infections in hospital environments still represent a significant public health problem. Before the COVID-19 pandemic, epidemiological data showed an increase in the number of infections caused by this pathogen suggesting the need for better preventive strategies. During the pandemic, the monitoring of water systems and the traceability of legionellosis cases were inadequate; therefore, a further increase in the number of *Legionella* infections is expected in the coming years. In this scenario, significant advancements have been obtained in the management and monitoring of *Legionella*. Overall, the data reported in the present manuscript can be summarized as follow:

- The precise characterization of *LP* clinical and molecular features is essential to identify potential virulence or drug resistance factors useful for the selection of the most effective antibiotic treatment;
- Disinfection strategies using chemicals, filters or UV lamps are essential in both water tanks, pipes and POU to reduce the risk of *LP* water contamination;
- The use of molecular high-sensitive diagnostic methods, such as ddPCR, besides the standard culture methods, could be useful to correctly diagnose legionellosis in suspected *LP* pneumonia.

To further corroborate the clinical validity of these advancements, further studies are needed to validate the diagnostic accuracy of novel diagnostic techniques and the efficacy of new treatment and disinfection protocols.

## Figures and Tables

**Table 1:** *L. pneumophila* virulence factors.

	Virulence Factors	Encoding Genes	Roles
Surface Proteins	Hsp60	*htpB*	Attachment in host cells, modulation of invasion and cytokine expression in macrophages [36]
	MOMP	*momps*	Activation of an alternative pathway of complement CR1 and CR3, phagocytosis [37]
	Mip	*Mip*	Penetration and replication in host cells [38]
Secretion systems	Type I Lss	*lssXYZABD locus*	Secretion of rtxA, attachment and penetration in host cells [34,59]
	Type II Lsp	*PilEL*	Secretion of other virulence factors [42]
	Type IV	*Lvh*	Conjunction, secretion of virulence factors, Legionella survival [43,60]
		*IcM/Dot*	Conjunction, transport and injection of DNA [48,49]
Two component system	CpxRA	*cpxA, cpxR, cpxRA*	Transcription of anti-stressor genes, regulation of IcM/Dot system effectors [57,58]

## Data Availability

The data described in the present manuscript are all available on PubMed NCBI.
